# Network Pharmacology and Natural Products in Modern Drug Discovery: Emerging Trends, Challenges, and Future Directions

**DOI:** 10.3390/ph19050653

**Published:** 2026-04-22

**Authors:** Alexander Panossian

**Affiliations:** Phytomed AB, Sjöstadsvägen 6A, 59344 Västervik, Sweden; ap@phytomed.se

The past decade has witnessed a paradigm shift in pharmacological research, moving from reductionist, single-target drug discovery toward systems-based, multitarget therapeutic strategies. This transition has largely been driven by advances in network pharmacology (NP), multi-omics technologies, and artificial-intelligence-based drug discovery [1,2,3,4]. The collection of recent publications in *Pharmaceuticals* (2025–2026) shown in Supplementary Table S1 reflects this transformation, highlighting the expanding role of natural products, traditional herbal medicines, and integrative pharmacology in addressing complex diseases (Figure 1) [3,4,5,6,7,8,9,10,11,12,13,14,15].

An expert community in China [2] has officially recognized NP under the guidelines of the Communist Party focused on modernizing the archaic theories of TCM through a holistic/integrative approach, since the reductionistic/differential approach based on another archaic theory pertaining to Galenic preparations is not always suitable for new-drug discovery due to multitarget effects, complex botanical combinations, and purified compounds [4]. Notably, both approaches are complementary and essential for achieving the desired goal [4].

This Special Issue includes 12 original articles and 2 reviews (Supplementary Table S1) on NP of botanicals from seven Asian countries predominantly implementing traditional medical systems (including China, Japan, South Korea, and Saudi Arabia) and Western countries following mainly conventional medicine paradigms (such as the USA, the UK, and Sweden), representing a variety of ethnic groups and political views. In this context, as declared by Sune K. Bergström, one of the Nobel Laureates in Medicine in 1982, “science does not know any national borders. The scientists of the world are forming an invisible network with a very free flow of scientific information—a freedom accepted by the countries of the world irrespective of political systems or religions….” [16].

These studies collectively emphasize multitarget mechanisms, computational drug discovery, experimental validation, and translational potential [17]. Moreover, they underscore the importance of systems-level understanding in treating multifactorial diseases such as neurodegeneration, inflammation, metabolic disorders, and infectious diseases. This editorial synthesizes the key findings and contributions of these publications, outlines recent developments in the field, identifies knowledge gaps, and proposes future research directions.

A major development across these studies is the transition from single-target drug discovery to multitarget network pharmacology. This approach reflects the complex nature of chronic diseases and supports the therapeutic use of multi-component natural products.

The review on Cordyceps and cordycepin highlights the fact that multiple signaling pathways—including PI3K–Akt, AMPK–mTOR, MAPK, and NF-κB—serve as mechanistic hubs underlying pleiotropic bioactivity. These pathways regulate immune responses, metabolism, and cellular resilience, supporting the notion that these compounds are adaptogenic [4].

The studies on traditional complex Botanical Herbal Product (BHPs) demonstrate multitarget mechanisms [18]:

- BHP Banhasasim-Tang, composed of eight botanicals and used to treat irritable bowel syndrome, was found to operate via TNF signaling and apoptosis pathways as central mechanisms, as validated through in vivo models [8];
- BHP Qingfei Tongluo Jiedu, composed of nine botanicals and used for the treatment of pneumonia, regulates macrophage polarization via the butyrate–GPR109A–MAPK pathway, demonstrating immunomodulatory activity [15];
- BHP XuanYunNing, composed of 10 botanicals and used for the treatment of Meniere’s disease, a rare inner-ear disorder characterized by symptoms such as vertigo and hearing loss, disrupts a pathological cycle driven by JAK-STAT signaling, inflammation, and metabolic dysfunction [9];
- BHP Dahuang Xiaoshi -M (DXT-M), composed of three botanicals and used as a hepatoprotective agent in the treatment of liver injuries, demonstrated significant effectiveness in ameliorating liver pathologies, correcting abnormal liver function biomarkers, and regulating the CYP/GST-ROS axis [12].

Monoherbal botanical products, such as *Gynostemma pentaphyllum* [13], *Artemisia scoparia* [7], *Aronia melanocarpa* [14], and *Vitis vinifera* berry oligomeric polytcianodins [10], contain many active phytochemicals and exhibit multitarget effects; for instance, *Gynostemma pentaphyllum* exhibits dual-pathway modulation capacity, involving inflammatory signaling and renin–angiotensin system regulation, useful for COVID-19 treatment.

In several studies in this collection, multi-omics analyses were employed to identify molecular mechanisms and biomarkers. Even purified compounds such as Schisandrin B [11] and cordycepin [4] demonstrate multitarget action. The review on Cordycepin highlights how systems-level network analysis complements reductionist molecular pharmacology, fostering a holistic understanding of adaptogenic mechanisms [4].

These findings illustrate the shift toward holistic pharmacological frameworks.

Another significant trend is the integration of computational predictions with experimental validation. Most studies combine network pharmacology tools, molecular docking, molecular dynamics simulations, and in vitro/in vivo validation. This complementary approach represents a methodological advancement, improving prediction reliability and translational potential.

The integration of multi-omics technologies—including transcriptomics, proteomics, metabolomics, and microbiomics—has further transformed natural-product research. These approaches allow comprehensive analysis of biological systems and identification of novel therapeutic targets [17]. Multi-omics integration in herbal medicine research enables the identification of pathway-level interactions, thereby facilitating the development of precision therapeutics [3].

The therapeutic areas covered in several of these studies center on inflammatory and infectious diseases. The study on Qingfei Tongluo Jiedu demonstrated immune regulation through macrophage polarization, providing a novel therapeutic approach for drug-resistant pneumonia [15].

Similarly, the study on *Gynostemma pentaphyllum* revealed dual-mechanistic pathways involving immunological and renin–angiotensin system regulation useful for COVID-19 therapy [13].

These findings highlight the growing importance of immunomodulatory natural products.

Natural products are attracting an increasing amount of attention for their potential role in treating neurodegenerative disorders. The bibliometric analysis of natural products used for treating cognitive disabilities demonstrated rapid growth in this research field, with an annual growth rate of 12.36% from 1971 to 2024 [6]. This bibliometric study on cognitive disabilities identifies research trends, highlights knowledge gaps, and suggests future directions. The authors identified Alzheimer’s disease as the dominant research focus, followed by oxidative stress, neuroprotection, and molecular-docking approaches. Emerging trends include ferroptosis, UPLC-Q-TOF-MS, and network pharmacology methodologies [6]. However, the study also highlighted several gaps, including the underrepresentation of autism spectrum disorders and traumatic brain injuries as well as limited clinical validation of computational findings. These findings underscore the need for increased research on various cognitive disorders.

Natural products also show promise in regard to metabolic and chronic diseases. The review on Cordyceps discusses the adaptogenic effects in stress-related and aging-related disorders through metabolic and mitochondrial regulation [4], identifying multitarget adaptogenic mechanisms, highlighting regulatory challenges, and proposing a resilience-based therapeutic category. This research supports the use of natural compounds for preventive medicine and resilience-based therapeutics.

One of these publications’ major conceptual contributions is their recognition of adaptogens as resilience-supporting agents [3,4]. The authors of the review on Cordyceps propose a new regulatory category for resilience-supporting physiological modulators, emphasizing homeostasis rather than disease-specific treatment [4].

This concept aligns with preventive medicine and personalized healthcare.

Another important concept is multi-component drug synergy [18]. Herbal formulations often contain multiple compounds acting synergistically.

The study on *Gynostemma pentaphyllum* demonstrates the complementary actions of flavonoids and saponins, reinforcing the concept of polypharmacology [13].

This approach may improve therapeutic efficacy and reduce adverse effects. Two studies in this Special Issue demonstrate that

- *Aronia melanocarpa* berries can effectively overcome 5-FU resistance in colorectal cancer cells by targeting the TLR3/NF-κB pathway [14];
- Oligomeric proanthocyanidins from French grape (*Vitis vinifera* L.) seed extract can overcome chemotherapy resistance by targeting the integrin pathway in individuals with hepatocellular carcinoma [10].

Recent innovations include artificial intelligence and machine learning, molecular docking and dynamics simulations, multi-omics integration, and systems biology approaches. These technologies and approaches are transforming natural-product drug discovery.

Despite significant advances, there are still several knowledge gaps [1,2,19]:

- *Limited Clinical Evidence*: Most studies remain preclinical, highlighting the need for randomized clinical trials.
- *Standardization Challenges*: Variability in herbal preparations complicates reproducibility.
- *Bioavailability Issues*: The review on Cordyceps highlights the importance of low systemic concentrations of cordycepin, suggesting indirect mechanisms and emphasizing the need for pharmacokinetic studies.
- *Mechanistic complexity*.

Future research should emphasize clinical translation, multi-omics integration, AI-driven drug discovery, precision medicine, and regulatory-framework development.

The publications in this Special Issue demonstrate rapid advancement in natural-product pharmacology driven by network pharmacology, computational approaches, and systems biology. These studies collectively support a shift toward multitarget therapeutic strategies for complex diseases.

Future research should emphasize clinical translation, mechanistic understanding, and regulatory harmonization to fully realize the potential of natural products in modern pharmacotherapy.

## Figures and Tables

**Figure 1:**
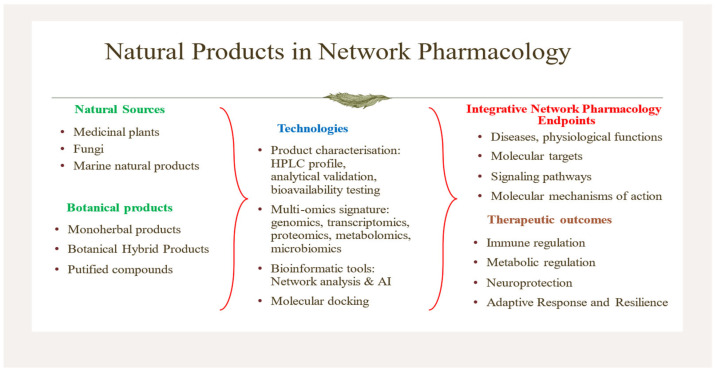
The natural sources of botanical products, technologies, integrative network pharmacology endpoints, and therapeutic outcomes covered in publications in this Special Issue.

## Data Availability

No new data were created or analyzed in this study.

## Supplementary Materials

The following supporting information can be downloaded at https://www.mdpi.com/article/10.3390/ph19050653/s1. Supplement S1 on the website. Table S1: Overview of the network-pharmacology studies in the Special Issue “Network Pharmacology of Natural Products, 2nd Edition”.
