# Supplementary material for: Network Pharmacology and Natural Products in Modern Drug Discovery: Emerging Trends, Challenges, and Future Directions
Source: Pharmaceuticals (Basel). 2026 Apr 22;19(5):653. doi: 10.3390/ph19050653 (PMC13209559; doi:10.3390/ph19050653)
Supplement: Supplementary file 1 [file pharmaceuticals-19-00653-s001.zip › pharmaceuticals-4261823-supplementary.pdf]

Table S1. Overview of network-pharmacology studies in Special Issue "Network Pharmacology of Natural Products, 2nd Edition..

Study, Year, Contry of origin	Dose/Concentration Main Bioactive Compounds	Disease or Physiological Function	Key Targets/ Protein Validated	Signaling Pathways	Type of Experimental Validation & Main Mechanistic Outcome
Panossion & Lemerond, 2025 [3], Sweden, USA	Ginseng, Rhodiola, Eleutherococcus, Schisandra, Ashwagandha; In a wide range of concentrations and dose-dependent reversal response in vitro, and in vivo	Stress-induced and aging-related disorders, Homeostasis supporting, adaptogenic activity, and antifatigue activity	PI3K, MAPK, JNK/MAPK10, PKC, p53, NF-kB; Nrf2, PKA, protein kinase A; PKB (Akt), protein kinase B; PLC, phospholipase C.	CRH- ACTH-BNDF induced adaptive stress-activated GPCR receptors mediated the activation of PI3K-Akt, MAPK, JNK/MAPK10, PKC, p53, NF-kB, nuclear factor-kappa B; Nrf2, nuclear factor E2-related factor 2 signaling pathways	The review highlights the importance of harmonized standards, transparent methodologies, and a balanced, evidence-informed approach to ensure consumers receive effective and safe botanicals. Future perspectives and proposed solutions include (i) establish internationally harmonized guidelines for evaluating botanicals based on their intended use (e.g., pharmaceutical vs. dietary supplement), incorporating traditional use data alongside modern scientific methods; (ii) encourage peer review and transparency in national assessments by mandating public disclosure of methodologies, data sources, and expert affiliations; (iii) create a tiered evidence framework that allows differentiated standards of proof for traditional botanical supplements versus pharmaceutical candidates; (iv) promote international scientific dialogs among regulators, researchers, and industry to develop consensus positions and avoid unilateral bans that may lack scientific rigor; (v) formally recognize adaptogens, a category of natural products for prevention of stress-induced brain fatigue, behavioral, and aging-related disorders
Panossian, 2026 [4], Sweden	Ophiocordyceps sinensis, Cordyceps militaris,, Cordycepin	Pleiotropic bioactivity, aging-related diseases Homeostasis supporting and adaptogenic activity	Inflammatory cytokines (TNF, IL-6, IL-1β), stress-activated kinases (MAPK1/3/8), survival pathways (AKT1),	PI3K–Akt, AMPK–mTOR, MAPK, NF-κB, apoptosis, and adaptive stress response signaling pathways, linking	O. sinensis, C. militaris, and cordycepin share a common adaptogenic mechanism for maintaining homeostasis of cellular and integrated biological system functions. The systems-level network analysis and reductionistic molecular ligand preceptor pharmacology provide complementary approaches for understanding the multi-target bioactivity of these fungi.

https://doi.org/10.3390/ph19050653

			mitochondrial regulators (FOXO3, HIF-1), apoptotic mediators (CASP3, BAX/BCL2), transcriptional co-regulators (CREBBP, EP300, FOXO3), and metabolic enzymes (IDH1, CYP19A1),	immune regulation and metabolic homeostasis.	The review clarifies conceptual and regulatory barriers to recognizing resilience-supporting interventions and informs future regulatory innovation.
Santiago-de-la-Cruz et al., 2025 [5], Mexoco	714 natural compounds, including 18 flavonoids have not been previously reported as senotherapeutics (3′,4′,7-trihydroxyisoflavone, daidzin, catechin, eriodictyol, auriculasin, pomiferin, 4′-O-methylalpinumisoflavone, tephrosin, 5,7-dihydroxy-3-(3-hydroxy-4-methoxybenzyl)-6-methoxychroman-4-one, calycosin-7-O-beta-D-glucoside, (R)-hesperetin, glycitin, jaceosidin, silybin, isosilybin A, eupafolin, and skullcapflavone II).	Ageing-related chronic degenerative diseases	p53, c-Fos, Cyclin D1, Trx, p21, AKT1, CDK1, NORE1, p65, c-Jun, and p38α	p53, ERK2, TGF-β1, IL-6, and AKT1 mediated signaling	Computational models, including Random Forest, Support Vector Machine, and K-Nearest Neighbours. Among 18 flavonoids, tephrosin, 5,7-dihydroxy-3-(3-hydroxy-4-methoxybenzyl)-6-methoxychroman-4-one, and isosilybin are particularly deserving of experimental verification for further development to improve their specificity, safety, and efficacy as senotherapeutics
Albratty et al., 2025 [6], Saudi Arabia, UK	Not specified	Cognitive Disabilities Alzheimer's disease, neuroprotection	Not specified	Not specified	Bibliometric overview, lack of experimental assessment.<br>The analysis underscores the dynamic and interdisciplinary nature of this field, highlighting areas for future exploration, particularly underrepresented cognitive disorders and novel therapeutic approaches.
Guo et al., 2025 [7], China	*Artemisia scoparia* (Yinchen) extract, consisting of 198 compounds including isochlorogenic acid C, isochlorogenic acid B, isochlorogenic acid A, and chlorogenic acid,<br>(50, 250, and 500 μg/mL extract	Neuroinflammation in Ischemic Stroke	TNF, STAT3, IL1B, AKT1 and SRC	Tumor signaling pathway, the processing of lipids and atherosclerosis, the mitogen-activated protein kinase (MAPK) signaling pathway, the calcium	The neurological outcomes were evaluated in a transient Middle Cerebral Artery Occlusion (tMCAO) rat model. Additionally, the changes in the inflammatory responses in both the ischemic brain and in LPS)-treated microglial cells were examined using real-time qPCR..Artemisia scoparia (Yinchen) extract was further found to exert neuroprotective effects against ischemia–reperfusion (I/R) injury and inhibit the production of inflammatory factors in tMCAO rats. Yinchen exerts an antineuroinflammatory effect

https://doi.org/10.3390/ph19050653

				signaling pathway, the cyclic guanosine monophosphate–protein kinase G (cGMP-PKG) signaling pathway, the inflammatory mediator regulation of transient receptor potential (TRP) channels, and the nuclear factor kappa B (NF-κB) signaling pathway.	on IS, and its constituents with high scores binding to five core targets contribute to this effect. This supports its potential as an anti-inflammatory agent for treating Ischemic Stroke
Choi et al., 2025 [8], Korea	BHP Banhasasim-tang (500 mg/kg in mice), composed of 8 botanicals: Coptidis Rhizoma(CR), Ginseng Radix (GR), Glycyrrhizae Radix et Rhizoma (GRR), Pinelliae Tuber (PT), Scutellariae Radix (SR), Zizyphi Fructus (ZF), Zingiberis Rhizoma (ZR), and Zingiberis Rhizoma Recens (ZRR). Eudesm-4(14)-en-11-ol (100 mg/kg), kanzonol T and elemol (100 mg/kg in mice)	Irritable Bowel Syndrome (IBS)	TNF-α PIK3CD PRKCD	TNF signaling and apoptosis as a key pathway.	The in vivo effects of eudesm-4(14)-en-11-ol, elemol, and Banhasasim-tang were evaluated in a zymosan-induced IBS mouse model. These findings provide preliminary mechanistic insight into the anti-inflammatory potential of BHSST in IBS. The integrated in silico and in vivo approaches support the contribution of specific components, such as eudesm-4(14)-en-11-ol, to its observed effects, warranting further investigation.
Jin, 2025 [9], China	BHP XuanYunNing tablets composed of 10 botanicals, including *Alisma plantago-aquatica* subsp. orientale Sam., *Atractylodes macrocephala* Koidz., *Wolfiporia cocos* (F.A. Wolf) Ryvarden & Gilb., *Pinellia ternate* (Thunb)Breit., *Ligustrum lucidum* Ait., *Eclipta prostrata* L., *Chrysanthemum morifolium* Ramat., *Achyranthes bidentata* Blume., *Citrus rticulata* Blanco., and *Glycyrrhiza uralensis* Fisch. In total, 125 compounds were identified, comprising 45 flavonoids, 29 terpenoids, 17 phenylpropanoids, 14 alkaloids, 13 organic acids, 3	Meniere's disease: a rare inner ear disorder characterized by symptoms such as vertigo and hearing loss	XuanYunNing downregulated IFNG, IFNGR1, JAK1, p-STAT3/STAT3, and AOX at both mRNA and protein levels	JAK-STAT signaling pathway as the central axis	XuanYunNing alleviates MD symptoms by disrupting a pathological cycle driven by JAK-STAT signaling, inflammation, and metabolic dysfunction. Key molecular targets and signaling pathways were further validated using RT-qPCR and Western blotting. XYN treatment reduced this area by 19% and significantly improved functional parameters, including the overall physiological condition in guinea pigs guinea pig model of endolymphatic hydrops.

	coumarins, 1 lignan, 1 quinone, and 2 others. Doses: 0.9, 1.8, and 3.6 g/kg) in guinea pigs.				
Noma et al., 2025 [10], Japan, China, USA	Oligomeric proanthocyanidins (OPCs) from French grape (*Vitis vinifera* L.) seed extract. IC50 values in Lenvatinib-resistant cell lines of 147.9 μg/mL for rHuh-7 and 162.0 μg/mL for rPLC.	Lenvatinib Resistance in Hepatocellular Carcinoma	OPCs and the combination treatment suppressed ITGA3 and the phosphorylation of EGFR and AKT.	ITGA3–EGFR–AKT signaling inhibition	OPCs can overcome chemotherapy resistance by targeting the integrin pathway, providing scientific evidence for their potential use as an adjunctive therapy for chemotherapy-resistant HCC
Gao et al., 2025 [11], China	Schisandrin B 20 μM and 40 μM Sch B groups (*in vitro*, AML-12 mouse liver cell line)	Metabolic associated fatty liver disease (MAFLD), formerly known as nonalcoholic fatty liver disease (NAFLD)	PPARγ-PCK1 and Caspase-3	**PPARγ-PCK1 and Caspase-3 Signaling Pathways**	Schisandrin B regulates lipid metabolism disorders in the MAFLD cell model. The activation of PPARγ-PCK1/Aspase is a key step that can effectively block fatty acid synthesis, improve fatty acid oxidation, and reduce lipid droplet aggregation in liver cells, thereby alleviating lipid metabolism abnormalities in the MAFLD cell model and inhibiting cell apoptosis.
Kong et al., 2025 [12], China	BHP Dahuang Xiaoshi -M (DXT-M), composed of 3 botanicals: *Rheum palmatum* rhizome, *Phellodendron chinense* cortex, *Gardenia jasminoides* fruit extracts ar doses of 5.4, 10, and 20 times the human equivalent dose in rats.	Liver injury, hepatoprotective	322 unique targets, including revealing 37 targets in the metabolite–reaction–enzyme–gene network. Particularly GSTM1, IL1B, GSTP1, MMP2, TNF, CYP2B6, CYP1A1, CYP1A2, and CYP3A4.	CYP/GST-ROS axis	DXT-M demonstrated significant effectiveness in restoring liver pathology, correcting abnormal liver function biomarkers, and regulating complement factors. The CYP/GST-ROS pathway served as both a shared regulatory axis and a transformation site for DXT-M's liver-protective effects.
Kim et al., 2025 [13], Korea, China	*Gynostemma pentaphyllum 59* flavonoids and saponins Dose is not specified	COVID-19	IL1B, IL6, TNF, ACE, and REN genes	Cytokine signaling, inflammatory responses, and the renin–angiotensin system.	Network analyses evaluated interactions of flavonoids and triterpenoid saponins with immunological, inflammatory, renin–angiotensin system, and host entry pathways. Molecular docking was performed to validate the binding affinities of rutin (−11.9 kcal/mol), NOS2–gypenoside LI (−11.6 kcal/mol), and ACE–gypenoside LI.
Duan et al., 2026 [14], Japan, USA	*Aronia melanocarpa* berry (ABE) extract, concentrations: 30, 60, 90, 120, and 150 μg/mL,	5-Fluorouracil Resistance in Colorectal Cancer	CD44, Nanog, Oct4 NF-κB; Toll-like receptor 3 (TLR3)	TLR3/NF-κB Signaling	ABE also reduced cancer stemness, as evidenced by reduced expression of CD44, Nanog, and Oct4. ABE

https://doi.org/10.3390/ph19050653

					effectively overcomes 5-FU resistance in CRC by targeting the TLR3/NF-κB signaling axis. This study highlights ABE as a safe, accessible, and promising adjunctive strategy to enhance therapeutic responses in 5-FU-resistant CRC.
Liu et al., 2026 [15], China	BHP Qingfei Tongluo Jiedu (QTJD), nine herbal components: *Morus alba* bark, *Lycium chinense* bark, *Polygonum cuspidatum* root, *Scutellaria barbata* root, *Prunus persica* semen, *Pheretima aspergillum* earthworm, *Perilla frutescens* fruit, *Descurainia sophia* semen, and *Glycyrrhiza uralensis* root. The low-dose group received QTJD 1.4 g dry extract/kg/day (4.6 crude drug/kg/day), the medium-dose group received QTJD 2.8 g dry extract/kg/day (9.2 crude drug/kg/day), and the high-dose group received QTJD 5.6 g dry extract/kg/day (18.4 crude drug/kg/day) in rats.	Mycoplasma pneumoniae pneumonia (MPP)	10 core targets, including JUN and Tumor Necrosis Factor (TNF), Mitogen-Activated Protein Kinase (MAPK), and Phosphoinositide 3-Kinase-Protein Kinase B. CD86, CXCL10, CD206, Arg-1, MRC-1, ERK1/2, p38, and JNK1/2	butyrate-GPR109A-MAPK pathway	Western Blot Analysis of MAPK, Mice alveolar-derived macrophages polarisation testing. QTJD effectively alleviates MPP by regulating macrophage polarization through the butyrate-GPR109A-MAPK pathway.

https://doi.org/10.3390/ph19050653
